# Applications of Optical Coherence Tomography in Optic Nerve Head Diseases: A Narrative Review

**DOI:** 10.3390/diagnostics15233001

**Published:** 2025-11-26

**Authors:** Mohamed M. Khodeiry, Elizabeth Colvin, Mohammad Ayoubi, Ximena Mendoza, Maja Kostic

**Affiliations:** 1 Department of Ophthalmology and Visual Sciences, University of Louisville, Louisville, KY 40202, USA; mmk591@miami.edu; 2 Bascom Palmer Eye Institute, Miami University Miller School of Medicine, Miami, FL 33136, USA; mxa1947@med.miami.edu (M.A.); xxm275@miami.edu (X.M.); 3 Department of Ophthalmology, University of Texas at Houston, Houston, TX 77030, USA; elizabeth.r.colvin@uth.tmc.edu; 4 Bascom Palmer Eye Institute, Ophthalmology Department, University of Miami Miller School of Medicine, Miami, FL 33136, USA

**Keywords:** optical coherence tomography, imaging, optic nerve

## Abstract

Optical coherence tomography (OCT) is a non-invasive imaging tool that is currently used in the evaluation and management of neuro-ophthalmic disorders. The detailed ability to visualize the optic nerve head, peripapillary retinal nerve fiber layer, and the macula, including the ganglion cell layer, allows for both qualitative and quantitative analysis of optic nerve diseases. This review covers the technical aspects of OCT and related imaging techniques in neuro-ophthalmology and discusses its use in common optic nerve head diseases such as optic disc drusen, optic disc coloboma, and elevated intracranial pressure. It also explores emerging OCT angiography applications in these disorders.

## 1. Introduction

The optic nerve head (ONH) is the intraocular portion of the optic nerve, where retinal ganglion cell axons converge and exit the eye to form the optic nerve, transmitting visual information to the brain. The ONH is anatomically composed of the neuroretinal rim, central cup, and the lamina cribrosa, a collagenous meshwork that provides structural support and is susceptible to biomechanical stress and remodeling, particularly in glaucomatous disease [1,2]. The ONH is traversed by the central retinal artery and vein and is surrounded by the peripapillary retinal nerve fibre layer (pRNFL), which is formed by the unmyelinated axons of retinal ganglion cells [3].

The ONH is supplied primarily by branches of the short posterior ciliary arteries, with venous drainage through the central retinal vein [4]. Surrounding the disc, the peripapillary retinal nerve fiber layer (pRNFL) contains unmyelinated RGC axons that converge toward the disc margin. Functionally, the ONH acts as a critical interface between the retina and the central visual pathways, maintaining axoplasmic flow and neural integrity [5]. Disruption of its structural or vascular homeostasis, as seen in glaucoma or ischemic optic neuropathies, leads to progressive RGC loss and irreversible visual field deficits [1].

Optical coherence tomography (OCT) is a commonly used tool within the field of ophthalmology to aid in detecting and monitoring retinal and optic nerve pathologies (Table 1) [6]. Advances in OCT technology through increased scanning speed, sensitivity, and resolution have transformed how clinicians monitor optic nerve and retinal diseases [7]. The most commonly used types of OCT are time domain OCT (TD-OCT), spectral domain OCT (SD-OCT), and swept-source OCT (SS-OCT). SS-OCT has the highest scanning speed (100,000–400,000 A-scans per second) out of the three types of OCT. SS-OCT and SD-OCT have the same imaging range, capturing the posterior cortical vitreous to the sclera [8]. However, SS-OCT has enhanced-depth imaging. TD-OCT imaged from the vitreoretinal interface to the retinal pigment epithelium (RPE) with a scan speed of 400 scans per second, making it inferior to the newer options available [8]. Furthermore, while many OCT-derived biomarkers, including pRNFL and ganglion cell–inner plexiform layer (GCIPL) thinning, primarily reflect axonal loss rather than direct ONH deformation, these changes remain highly relevant to optic nerve head pathology as they represent downstream structural consequences of ONH disease processes.

OCT angiography (OCT-A) is a complementary imaging technique to OCT based on the variable backscattering of light from retinal vasculature and neurosensory tissue, creating non-invasive motion-contrast images [9]. OCT-A enables analysis of the optic nerve head vasculature in order to assess for perfusion abnormalities [10]. These images enable clinicians to analyze depth-resolved information related to disease processes like diabetic retinopathy, glaucoma, ischemic optic neuropathies, and more [9]. OCT and OCT-A have become invaluable in the clinical setting to manage optic nerve pathologies. This paper will review the characteristic findings of specific pathologies utilizing OCT/OCT-A analysis to better aid in their diagnosis.

## 2. OCT in Glaucoma

**Figure 1:**
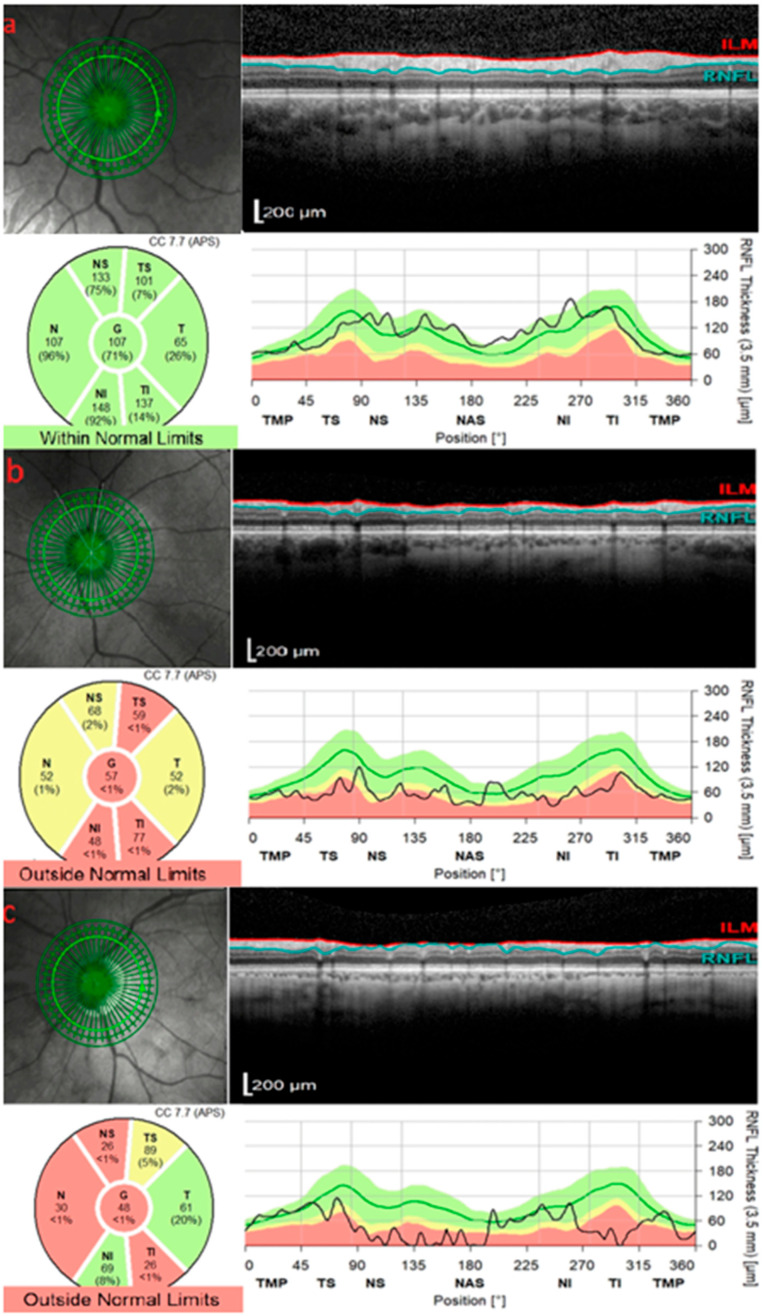
Spectral-domain optical coherence tomography (SD-OCT) analysis of the peripapillary retinal nerve fiber layer (RNFL) thickness in different eyes. (**a**) Non-glaucomatous emmetropic eye demonstrating RNFL thickness values within normal limits. (**b**) Glaucomatous emmetropic eye showing diffuse RNFL thinning, predominantly in the superior and inferior quadrants, classified as outside normal limits. (**c**) Glaucomatous myopic eye with pronounced RNFL attenuation and peripapillary thinning, also outside normal limits. ILM = internal limiting membrane; RNFL = retinal nerve fiber layer; TMP = temporal; TS = temporal–superior; NS = nasal–superior; NAS = nasal; NI = nasal–inferior; TI = temporal–inferior. Adapted from [25]. Licensed under a Creative Commons Attribution 4.0 International (CC BY 4.0) License. © 2024 Zawadzka et al. [25].

Glaucoma is a leading cause of irreversible blindness worldwide [11]. It is characterized by progressive optic neuropathy, characterized by retinal ganglion cell loss and corresponding visual field defects [12]. OCT plays a central role in glaucoma diagnosis and management by objectively measuring structural damage in three key areas: the peripapillary retinal nerve fiber layer (RNFL), the macula (including the ganglion cell complex [GCC] and the ganglion cell–inner plexiform layer [GCIPL]), and the optic nerve head (ONH) (Figure 1). OCT is superior to other imaging modalities in detecting and monitoring RNFL and GCIPL changes in glaucoma, as it provides micrometer-scale quantitative measurements and often identifies structural loss before visual field defects are apparent. The decision of how frequent OCT should be done in glaucoma patients is based on clinical judgment and glaucoma severity [13,14]. Peripapillary RNFL thickness is one of the most established OCT biomarkers for glaucoma, with studies demonstrating an area under receiver operating characteristics curve of around 0.9 [15]. Prior studies have shown that the RNFL in the superior and inferior quadrants may be more susceptible to glaucomatous damage [16,17,18]. For example, Rao et al. reported that among RNFL quadrants, the inferior quadrant demonstrated the highest diagnostic performance for detecting early glaucoma, with an AUC of 0.821, sensitivity of 58.2% and specificity of 95% [16].

When it comes to different types of glaucoma, distinct patterns of RNFL thinning have been observed across subtypes of open-angle glaucoma (OAG). Baniasadi et al. utilized SD-OCT to compare the different patterns of RNFL thinning in 4 subtypes of open-angle glaucoma (OAG): primary OAG (POAG), normal tension glaucoma (NTG), pseudoexfoliation glaucoma (PXG), and pigmentary glaucoma (PDG). Compared to PXG, POAG and NTG demonstrated significantly more RNFL thinning in the inferior and inferior-temporal sectors (*p* = 0.002–0.018 and *p* = 0.006, respectively). In contrast, PDG showed greater thinning in the superior and superior-nasal sectors compared to POAG, NTG, and PXG (*p* = 0.009, 0.003, and 0.009, respectively) [19].

Over the past decade, increasing attention has been directed toward the macula, which contains nearly 50% of all retinal ganglion cells [20,21]. Minimum GCIPL has shown diagnostic accuracy comparable to RNFL and ONH metrics, with some studies identifying it as the most sensitive macular measure for early glaucoma detection [22,23]. However, a recent systematic review of 34 studies comparing macular and RNFL parameters across multiple SD-OCT platforms concluded that RNFL measurements remain slightly more accurate for diagnosing manifest glaucoma, although the differences were modest [24].

## 3. OCT in Anterior Ischemic Optic Neuropathy

Anterior ischemic optic neuropathy (AION) is caused by insufficient blood flow to the ONH and is classically divided into two categories: arteritic AION and non-arteritic AION (NAION) [26]. Arteritic AION is most commonly caused by giant cell arteritis, while NAION is caused by a state of hypoperfusion [26,27]. OCT has emerged as a useful tool in evaluating AION by documenting optic disc edema acutely and, subsequently, atrophy.

NAION typically presents with acute onset of painless vision loss, optic edema, and a relative afferent pupillary defect [28]. OCT is a valuable tool for detecting acute optic disc edema in NAION, particularly when clinical signs are subtle. OCT typically reveals peripapillary RNFL thickening in the acute phase, followed by progressive thinning over the subsequent months [29]. The degree of RNFL thinning might correlate with functional outcomes including visual acuity and visual field loss [30]. Thus, serial imaging allows for objective monitoring of RNFL thinning as the disease progresses, providing reassurance when the clinical course follows an expected trajectory. GCIPL has also been shown to be a valuable marker for assessing neuronal loss in NAION. Unlike RNFL, GCIPL thinning becomes detectable as early as one month after onset and continues through the subacute phase, stabilizing by six months [31]. This early loss reflects underlying ganglion cell degeneration and often precedes measurable RNFL thinning, which may initially be obscured by axonal edema. Moreover, GCIPL thinning correlates strongly with visual acuity and visual field deterioration, offering a reliable structural indicator of functional impairment [32].

Comparatively, arteritic AION (AAION) tends to cause even more profound damage to the optic nerve. Clinically, AAION often presents with more severe vision loss than NAION and a pale, swollen optic disc [33]. In contrast to NAION, OCT has not been as widely utilized in AAION. Pellegrini et al. compared the choroidal vascularity index (CVI) among patients with arteritic AION and non-arteritic AION using SD-OCT. Patients with arteritic AION had significantly lower macular and peripapillary CVI than both NA-AION patients (67.17 ± 2.35 vs. 69.66 ± 4.18, *p* = 0.048; 63.51 ± 3.29 vs. 67.67 ± 3.07, *p* < 0.001). After adjusting for age, peripapillary CVI differences remained significant (*p* < 0.001), suggesting that CVI may serve as a useful biomarker to distinguish arteritic from non-arteritic AION [34].

OCT-A can also play a role in the management and monitoring of AAION and NAION. Findings include reduced radial peripapillary capillaries (RPC) in the acute phase, and progressive vessel reduction in the chronic phase [35,36]. Vessel density in the optic nerve head and peripapillary region often correlates with the extent of RNFL thinning and visual field impairment. Pierro et al. reported that eyes with AAION demonstrated significantly lower vessel density (VD) and vessel tortuosity (VT) in both the radial peripapillary capillary (RPC) and superficial capillary plexus (SCP) layers compared to non-arteritic AION and controls. In affected visual field quadrants, AAION eyes showed reduced VD in the RPC (0.29 ± 0.04) and SCP (0.27 ± 0.04) compared to NAION (0.36 ± 0.01 and 0.33 ± 0.04, respectively). VT was also markedly decreased in AAION-affected quadrants in both the RPC (3.8 ± 0.4 vs. 4.6 ± 0.7) and SCP (2.8 ± 0.8 vs. 3.4 ± 0.5) compared to NAION. These findings highlight that AAION causes more severe microvascular damage than NAION, with VD serving as a good marker of localized impairment and VT providing a sensitive global indicator of vascular dysfunction [37].

## 4. OCT in Inflammatory Optic Neuropathies

Optic neuritis (ON) is classified as an inflammation of the optic nerve most commonly seen in young adults and frequently associated with demyelinating diseases such as multiple sclerosis (MS). Less common causes include infectious and systemic diseases. Common symptoms include reduced or blurred vision, pain that worsens with eye movement, and dyschromatopsia. If the ON is unilateral, there will be a relative afferent pupillary defect on examination.

**Figure 2:**
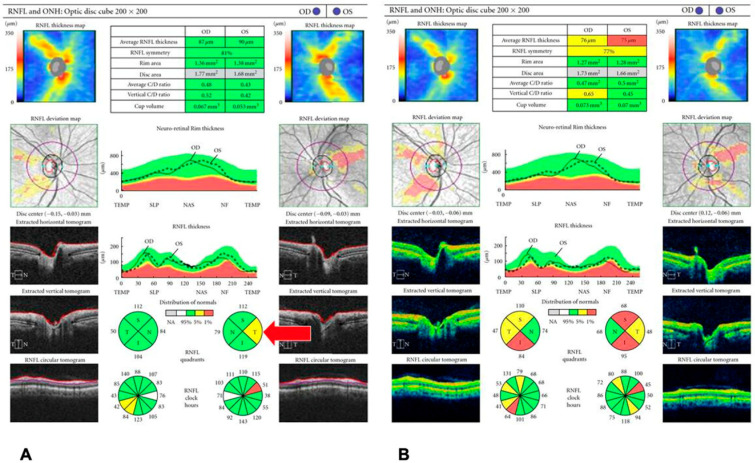
Spectral-domain optical coherence tomography (SD-OCT) findings in a 26-year-old woman with multiple sclerosis–related optic neuritis. (**A**) Baseline images show relatively preserved global average retinal nerve fiber layer (RNFL) thickness (OD: 87 μm; OS: 90 μm) with subtle temporal thinning in the left eye (arrow). (**B**) Follow-up images 2.5 months later demonstrate progressive global RNFL atrophy (OD: 76 μm; OS: 75 μm), corresponding to optic disc pallor and visual recovery to 20/20 in both eyes with mild residual color vision deficit. Adapted from [40]. Licensed under a Creative Commons Attribution (CC BY) License.

MS is the most common systemic condition associated with optic neuritis, and in many cases, ON may be the initial manifestation of the disease. In MS-related ON, inflammation and demyelination of the optic nerve can result in transient or permanent visual impairment. OCT plays a critical role in assessing axonal loss in these patients, with longitudinal measurements of the RNFL and GCIPL serving as reliable biomarkers of neurodegeneration in both clinically affected and unaffected eyes (Figure 2). While MRI is essential for detecting acute inflammation and lesion localization, OCT is uniquely valuable for monitoring chronic neuroaxonal injury and subclinical progression, which may not be captured by MRI or visual evoked potentials. OCT changes follow a distinct time course: during the acute phase, RNFL thickness is often elevated due to axoplasmic stasis, making it unreliable for early assessment of axonal loss. Detectable RNFL thinning typically emerges 2 to 3 months post-onset, while GCIP thinning occurs earlier and serves as a more sensitive and reliable marker of retrobulbar neuroaxonal injury [38]. In terms of quantification, Petzold et al. conducted a meta-analysis and found that the most significant and consistent retinal changes in MS involve thinning of the peripapillary RNFL and the macular GCIPL. Compared to controls, MSON (MS-associated optic neuritis) eyes showed substantial RNFL thinning (−20.10 μm) and GCIPL thinning (−16.42 μm), while MSNON (MS without optic neuritis) eyes also exhibited significant but lesser thinning (−7.41 μm RNFL; −6.31 μm GCIPL). Mild thickening of the inner nuclear layer (INL) was observed in MSON eyes, suggesting possible inflammatory activity [39].

Neuromyelitis optica (NMO) is an autoimmune astrocytopathy primarily targeting aquaporin-4 water channels, leading to recurrent episodes of optic neuritis and transverse myelitis [41]. Compared to multiple sclerosis, optic neuritis in NMO is typically more severe, often bilateral, and associated with poorer visual recovery. Similar to MS related-ON, OCT often shows a similar pattern of RNFL and GCIPL thinning. However, NMO ON thinning is generally more severe, which may help in differentiating the two pathologies [42]. The superior and inferior quadrants are preferentially affected in NMO ON, whereas MS ON typically targets the temporal quadrant. These patterns may reflect differences in axonal vulnerability and disease pathophysiology. Unlike MS, NMO eyes without a clinical history of ON generally show preserved RNFL thickness, indicating that subclinical ON is uncommon [43,44]. However, some studies have reported GCIPL thinning in these eyes, potentially due to underrecognized mild ON. In a comparative study involving 26 NMO spectrum patients with ON, OCT revealed significantly greater RNFL thinning (mean: 63.6 µm) than in MS ON eyes (88.3 µm) and healthy controls (102.4 µm), with a first ON episode causing approximately 24 µm more RNFL loss in NMO than MS. Similar reductions were observed in macular volume, and visual impairment was more severe in NMO ON eyes [45].

Neuroretinitis is another type of inflammation of the anterior optic nerve and peripapillary retina. It is most commonly caused by cat-scratch disease, which is an infection with the Bartonella henselae bacterium [46]. It classically presents as optic disc edema and a delayed appearance of a macular star (radial pattern of hard exudates in the outer plexiform layer). OCT reveals a range of distinct findings that support early diagnosis and disease monitoring. Initial signs include flattening of the foveal contour, accumulation of subretinal and intraretinal fluid, and hyperreflective exudates within the outer plexiform layer [47]. OCT may also reveal cell collections in the vitreous just anterior to the optic disc before they become apparent on slit-lamp biomicroscopy [48]. Recognizing this feature is particularly important in ambiguous cases, as it helps distinguish inflammatory or infectious causes of disc swelling from non-inflammatory conditions like papilledema or ischemic optic neuropathy. Retinal exudates within the outer plexiform layer may be identified on OCT prior to becoming clinically apparent on fundoscopic examination as the macular star [46]. Few studies have assessed OCT-A findings in neuroretinitis. In one case series of presumed diffuse unilateral subacute neuroretinitis (DUSN), OCT-A revealed decreased vascular perfusion in the superficial and deep capillary plexuses (SCP and DCP), while the choriocapillaris remained unaffected. One patient also demonstrated reduced vascular density in the optic disc and radial peripapillary capillary plexus. These findings suggest inner retinal vascular compromise, likely due to direct retinal damage or toxic effects from the nematode, and highlight OCT-A’s utility in identifying structural and vascular alterations in DUSN not evident on fluorescein angiography [49].

## 5. OCT in Optic Disk Neovascularization

Pathologic neovascularization of the optic disc (NVD) is a finding typically associated with ischemic retinal conditions, such as proliferative diabetic retinopathy or ischemic central retinal vein occlusion. Traditionally, fluorescein angiography (FA) has been the gold standard for detecting disc neovascularization. However, OCT-A now provides a non-invasive alternative to visualize these aberrant new vessels on and around the optic disc. A retrospective study involving 169 eyes assessed the utility of OCT-A and various OCT-A-based methods for detecting NVD. Compared to ultra-widefield color fundus photography (UWF-CFP), which detected NVD in only 34.91% of eyes, OCT and OCT-A each detected NVD in 59.76% of cases, while fluorescein angiography (FA) identified 62.72% [50].

In eyes with proliferative diabetic retinopathy (PDR), OCT-A enabled clear visualization of NVD, even in cases where FA was limited by excessive leakage. In one patient, OCT-A revealed dense, disorganized neovascular structures overlying the optic disc that were obscured on FA due to intense leakage. Following intravitreal anti-VEGF therapy, OCT-A demonstrated a marked reduction in NVD area by 2 weeks, with continued regression observed at 4 weeks. Despite initial improvement, OCT-A at 8 weeks showed enlargement of vessel diameters and an increase in irregular microvasculature, indicating recurrence of NVD [51]. These changes were quantitatively tracked using OCT-A, confirming its value not only for detecting NVD but also for monitoring therapeutic response and disease recurrence over time.

## 6. OCT in Papilledema and Pseudopapilledema

In the clinical setting, differentiating papilledema from pseudo-papilledema can be difficult but necessary for appropriate management and prognosis. Pseudopapilledema is an optic disc anomaly with nerve elevation without actual underlying swelling [52]. This is in contrast to papilledema, which is caused by an elevated intracranial pressure that extends into the peri-optic subarachnoid space [53]. Typically, the diagnosis of papilledema warrants further testing that could be invasive in some situations. Therefore, OCT can aid in the accurate diagnosis. When comparing other imaging modalities, OCT outperforms fundus photography and ultrasound in evaluating optic disc edema and distinguishing true papilledema from pseudopapilledema. OCT provides objective measurements of RNFL thickness and optic nerve head volume, and EDI-OCT can identify buried optic disc drusen, a key feature of pseudopapilledema, with higher sensitivity and specificity than other modalities [54].

**Figure 3:**
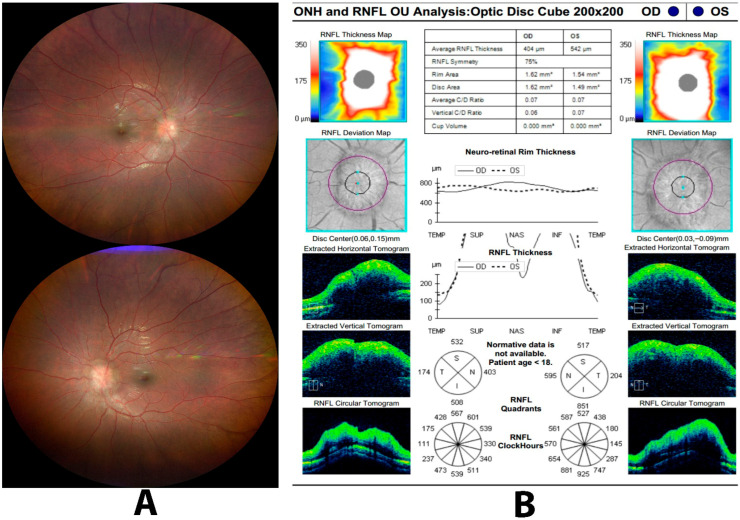
Fundus photograph (panel (**A**)) and optical coherence tomography of a patient with papilledema due to idiopathic intracranial hypertension (IIH). Fundus images demonstrate bilateral optic disc swelling with blurred disc margins and obscuration of peripapillary vessels, suggestive of true optic disc edema. OCT reveals marked thickening of the peripapillary retinal nerve fiber layer (RNFL) in all quadrants, particularly superiorly and inferiorly, with associated elevation of the optic nerve head on (**B**)-scan. These findings are consistent with papilledema.

Peripapillary RNFL thickness is a useful parameter for distinguishing papilledema from pseudopapilledema (Figure 3) [10,55,56,57,58]. Thickening of the RNFL in all four quadrants of the nerve is more indicative of papilledema versus an average RNFL thickness, which lends towards pseudo-papilledema; however, these findings may depend on the severity of the disease. Some limitations of utilizing the mean RNFL thickness as a diagnostic criterion for the diagnosis of papilledema include no normative values for pediatric patients (<18 years old), no normative adjustments for high myopes, and artifact on imaging [53,59].

The selected threshold of RNFL thickness may play a role in the diagnostic accuracy of papilledema [52,55,56,57,58]. Studies showed that the nasal sector of the RNFL possibly provides the most significant diagnostic information. However, there is no standardization of the definition of an abnormal RNFL thickness among various TD and SD-OCT devices [52]. Using TD-OCT, Johnson and associates [58] found a sensitivity of 80% and a specificity of 70% of true disc edema when the nasal RNFL thickness was greater than 86 μm, with an area under the ROC curve (AUC) of 0.86. Lee and colleagues [55] used a criteria of 78 μm, reporting a sensitivity of 80% and a specificity of 47%. These findings are comparable to Sarac et al. [56] who utilized a lower nasal RNFL thickness of >74.5 μm, resulting in a sensitivity and specificity of 92% and 47%, respectively. While these studies demonstrate a useful starting point for clinical differentiation, the moderate sensitivity suggests that early or mild cases of optic disc edema may still be missed, limiting its reliability as a standalone diagnostic threshold. Therefore, SD and TD-OCT supplement but not entirely replace the ophthalmic examination [53].

Fluorescein angiography (FA) has been considered the gold standard for diagnosing papilledema as it shows leakage from the optic nerve if swelling is present [53], but there is limited utility when evaluating the optic nerve head capillary plexus [10,60]. Recent studies have analyzed the effectiveness of utilizing OCT-A as a diagnostic tool for papilledema. In a study that evaluated OCT-A of 46 eyes of idiopathic intracranial hypertension (IIH) patients, OCT-A was reported to be effective for visualizing and quantifying the peripapillary retinal vasculature. They found skeletonized peripapillary vessel (SVD) density significantly decreased with increasing papilledema grade (R = 0.512; *p* < 0.001). SVD also significantly correlated with retinal nerve fiber layer thickness (R = 0.336; *p* = 0.022) and ganglion cell layer thickness (R = 0.408; *p* = 0.006), supporting the potential clinical utility of OCT-A as a non-invasive marker for disease severity and early optic nerve damage in papilledema [10].

## 7. OCT in Optic Pits

**Figure 4:**
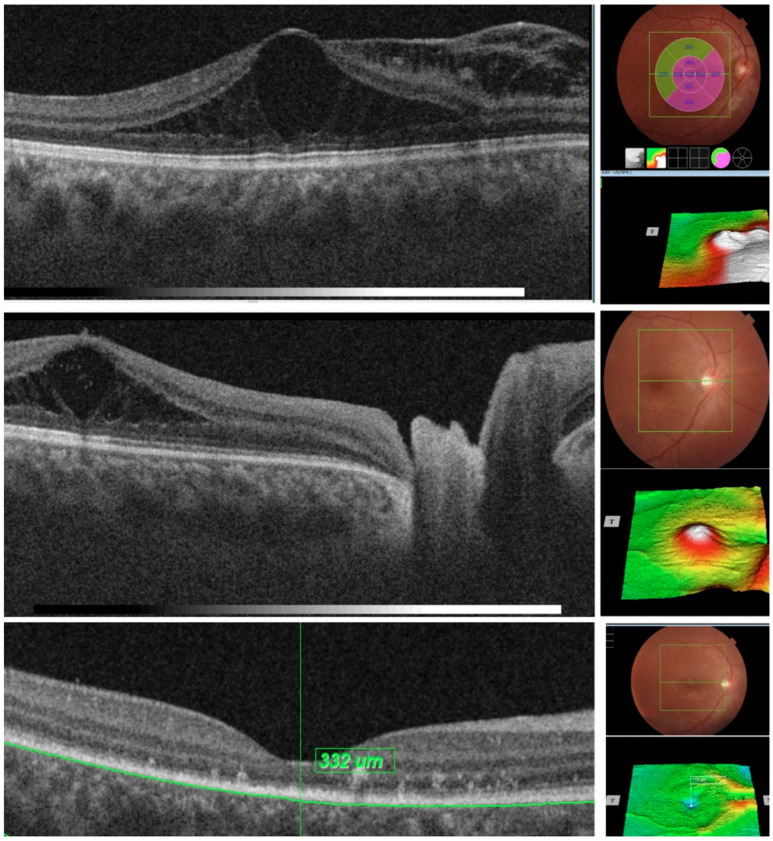
Sequential optical coherence tomography (OCT) scans showing progressive resolution of optic-disc-pit-associated maculopathy after a minimally invasive surgical approach. Initial scans demonstrate subretinal fluid with schitic separation (**top**), followed by gradual resolution of intraretinal cystoid changes (**middle** and **bottom**). Image adapted from [69].

Optic Pits (OP, also known as optic nerve pits, optic disc pits, or optic holes) are congenital defects defined by the herniation of dysplastic retinal tissue into an excavation that often extends into the subarachnoid space through a defect in the lamina cribrosa [61]. The incidence of OP is reported to be 1:1000 and 10–15% of the cases are bilateral [62,63]. On examination, these pits are identified by their small, oval-shaped depression in the optic disc, which can allow for fluid accumulation in the subretinal space [61]. OP is commonly noted in the temporal aspect of the optic nerve [64,65]. Patients with OP commonly suffer from serous macular detachments; however, the underlying pathogenesis is unclear, and the treatment of OP maculopathy is controversial [66]. Previous OCT technology, like TD-OCT, was unable to penetrate the deeper tissues of the retina, like the choroid and subarachnoid space, necessary for the evaluation of OP maculopathy. With the newer advances in OCT technology, these structures can now be visualized to progress the understanding of the pathogenesis and treatment of OP (Figure 4).

Enhanced depth imaging (EDI)-OCT was utilized by Margolis and Spaide [67] to visualize and measure the entirety of the choroid, allowing for further analysis of the relationship between age and choroidal thickness. Ohno-Mastui et al. [68] utilized SS-OCT to evaluate OP. The SS-OCT showed the entire pit from opening to bottom in twelve of the sixteen eyes with an average depth of 909 ± 449.5 μm. The most common OCT finding within their study was a defect in the lamina cribrosa at the site of the OP and a herniation of nerve tissue into the OP. In addition, SS-OCT analysis showed that the shape of the OP varied depending on the depth of the pit. The pits were also deeper within retinal tissue along the temporal border [68]. Therefore, the ophthalmic exam findings are only a surface-level representation of the actual impact OP has on the underlying retinal structures.

## 8. OCT in Optic Disc Coloboma

Optic disc coloboma is a congenital disease that is similar to optic disc pits in that they are both related to the same group of excavated disc anomalies [68,70]. A coloboma occurs due to a failure of the embryonic ocular fissure to close, but its underlying pathogenesis is not well understood [71]. Clinically, optic disc coloboma is seen as a sharply delimited, glistening white, bowl-shaped excavation that occupies an enlarged optic disc [68]. Colobomas are typically present along the inferonasal aspect of the eye due to the location of the embryonic fissure [71]. While the coloboma itself can impact vision, OCT is vital to monitor for choroidal neovascularization, a rare but visually devastating complication [62].

Jeng-Miller et al. [62] conducted a review of various congenital anomalies of the optic disc. They noted that there are far fewer studies analyzing disc colobomas and their findings on OCT. Ohno-Mastui et al. [68] utilized SS-OCT to evaluate seven eyes with optic disc colobomas. Only three eyes produced high-quality OCT images to enable deep structure analysis. Due to the irregular surface within the colobomatous area, it was difficult to obtain good scans. In the eyes imaged, SS-OCT showed that the optic nerve head was deeply excavated posteriorly. In addition, the scleral fibers in the colobomatous area were sparse and separated with random orientations [68]. SD-OCT imaging of disc colobomas demonstrates sclera immediately below the retina corresponding to the adjacent choroidal coloboma [63,72,73].

OCT-A analysis of disc colobomas showed the absence of the radial peripapillary microvascular network at the defect level [63,72]. This finding is similar to OP, but it is very distinct from the dense network found in morning glory anomaly. Therefore, these findings suggest that colobomas and OP are on a similar spectrum [63]. Further studies are needed to examine the OCT findings associated with colobomas to accurately diagnose and manage patients suffering from visual impairment.

## 9. OCT in Optic Nerve Head Drusen

Optic nerve head drusen (ONHD) are benign congenital anomalies of the optic disc that may be due to the accumulation of acellular deposits of mucoprotein, mucopolysaccharides, and calcium in the prelaminar part of the optic nerve. The prevalence of ONHD ranges from 0.4 to 3.7% of the overall population, with most of the cases occurring bilaterally (60 to 91% of cases) [74]. ONHD possibly develops as a result of impaired axonal metabolism with mitochondrial calcification, leading to disruption of axonal cells and release of mitochondria in the extracellular space with more calcium deposition in the mitochondria that eventually form the drusen [75]. A narrow scleral canal could predispose to the development of ONHD through compression of axonal fibers, leading to their degeneration and collection of degenerative calcified mitochondria [76]. On the contrary, some studies found that scleral canals are larger in patients with ONHD compared to normal population [77]. EDI-OCT is the preferred imaging technique, offering the highest sensitivity and accuracy for identifying and characterizing optic nerve head drusen compared to B-scan ultrasonography, fundus photography, and fundus autofluorescence. EDI-OCT enables detailed visualization of drusen morphology and location, supporting its role as the gold standard for drusen detection [78].

There are 2 types of ONHD: superficial and buried. Superficial ONHD appears as hardened yellow deposits on the surface of the optic nerve. On the other hand, on fundoscopic exam of patients with buried ONHD, optic nerve head appears elevated with an irregular, scalloped border without obscuration of the peripapillary vessels. The buried drusen are more frequent in children; they may become more visible with age and increase in number and size [79].

SD-OCT has a limited depth of penetration, so its role in the assessment of ONHD is limited. SS-OCT utilizes a tunable laser at speeds of 100,000 to 236,000 A-scans/sec, allowing deeper tissue penetration roughly double that of spectral-domain optical coherence tomography (SD-OCT), higher imaging speed, higher detection efficiencies, and better imaging range. The advantages of SS-OCT make it a better option than SD-OCT for imaging of ONHD [80].

EDI-OCT has its highest sensitivity near the inner sclera. Thereby, it is more accurate in the imaging of ONHD, especially the buried subtype. EDI-OCT can provide more information about the extent of disc drusen better than FFA or B-scan ultrasonography. In a cross-sectional comparative study, Merchant et al. found that EDI-OCT was more sensitive in detecting ONHD compared to B-scan ultrasonography. The study defined ONHD as either hyporeflective areas surrounded by hyperreflective borders or collections of hyperreflectivity without the core. When disc drusen were visible on dilated optic disc photographs, both EDI-OCT and B-scan ultrasonography detected the ONHD. However, in 25 eyes with suspected ONHD, EDI-OCT detected drusen in 17 eyes compared to B-scan, which detected drusen in only 7 eyes [81].

**Figure 5:**
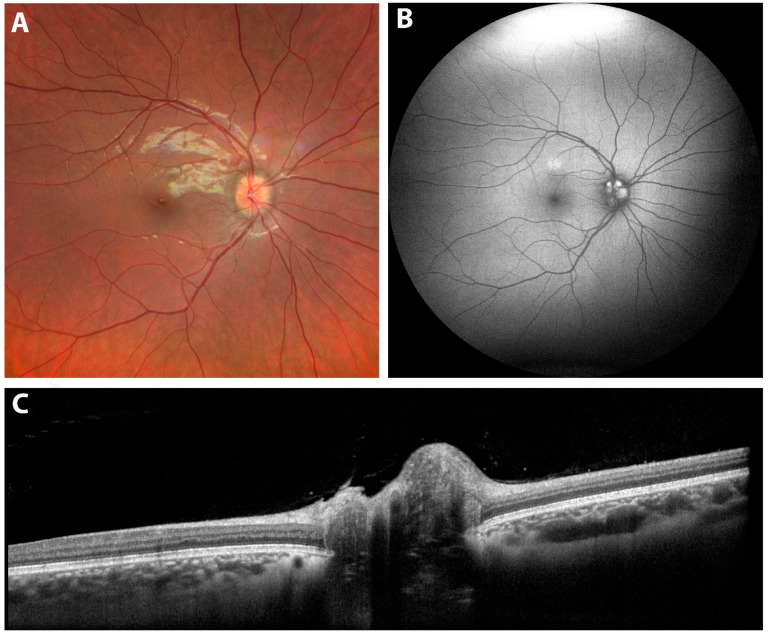
Fundus photo panel (**A**), fundus autofluorescence panel (**B**) and optic coherence tomography panel (**C**) of optic nerve head drusen. Panel (**A**) shows a crowded optic disc with irregular elevation and visible yellowish refractile deposits consistent with superficial drusen. Panel (**B**) demonstrates hyperfluorescence over the optic nerve heads. Panel (**C**) (OCT B-scan) reveals a signal-poor lesion with hyperreflective margins.

On EDI-OCT, ONHD always appears above the lamina cribrosa as a signal-poor core that it is often surrounded by a hyperreflective margin most prominent superiorly (Figure 5). ONHD may also present as conglomerates, with hyperreflective disrupted margins within the signal-poor core, so they possibly represent the fusion of smaller drusen. Although normal retinal blood vessels can be mistaken as ONHD due to a hyporeflective core, blood vessels can be distinguished by having anterior and posterior hyperreflective bands, and a “figure of eight” configuration in cross-section due to the arteriole-venule pairing. In addition, a review of other adjacent B-scan is helpful in the differentiation between retinal blood vessels and ONHD [82].

Peripapillary hyperreflective ovoid mass-like structures (PHOMS) are sometimes detected on EDI-OCT scans of ONHD. Some studies previously hypothesized that PHOMS are early or variant types of ONHD [83,84]. The characteristics of PHOMS—unlike ONHD—that argue this theory are the following: they lack the hyporeflective core, do not autofluoresce, can be seen in patients with papilledema, are not visible on ultrasonography, and are located in the peripapillary region of the optic disc. Histopathological studies suggested that PHOMS might correspond to the lateral bulging or herniation of distended axons into the peripapillary retina [82,85,86,87]. Hyperreflective bands (HRBs) are hyperreflective horizontal lines noticed in deep layers of the optic nerve head. HRBs are sometimes present in eyes with ONHD or in healthy eyes of patients with contralateral ONHD. Exact histopathology of HRB is not fully understood yet. Some studies suggest that hyperreflective bands may be a sign of early drusen [81,88].

Traber et al. used EDI-OCT to describe three morphologic types of ONHD: peripapillary hyperreflective ONHD, granular hyperreflective ONHD, and confluent hyporeflective ONHD. The confluent type was reported to be commonly associated with worse visual fields [84]. EDI-OCT is of paramount importance to understand the relationship between drusen and RNFL. Sato et al. found a significant negative correlation between the diameter of disc drusen and RNFL thickness. In addition, there was a significant positive correlation between the disc drusen diameter and the number of sectors of thinned RNFL. The presence of drusen within the optic canal was also associated with thinner RNFL [88].

OCT-A can provide a method to analyze the flow changes in patients with ONHD. Gaier et al. noted in a patient with bilateral superficial ONHD that OCT-A showed focal attenuation of superficial capillary plexus overlying the ONHD, in addition to a focal thinning of the RNFL correlating with the distribution of the ONHD [89]. In a study that evaluated 19 eyes with ONHD, eyes with ONHD had significantly lower flow index (0.09 ± 0.01 vs. 0.10 ± 0.01; *p* = 0.021) and vessel density (85.6% ± 0.05 vs. 90.7% ± 0.03; *p* = 0.001) compared to controls. OCT-A parameters were significantly correlated with GCC thickness (flow index: r = 0.545, *p* = 0.016; vessel density: r = 0.506, *p* = 0.027), but not with RNFL thickness (*p* > 0.05), suggesting that OCT-A may reflect early microvascular compromise independent of RNFL loss [90].

Buried ONHD could be difficult to differentiate from early papilledema, so OCT can provide additional clues to the history and clinical examinations. EDI-OCT is highly sensitive in discrimination between ONHD and optic disc edema. Choroidal wrinkles and folds are commonly seen in papilledema but not in ONHD [53]. In some eyes with papilledema, the angulation of Bruch’s membrane towards the vitreous is present, but it is not reported in ONHD. RNFL analysis has shown increased thickness, more nasally, in papilledema compared to ONHD [91]. In one study, an average RNFL thickness < 116 µm on SD-OCT demonstrated a sensitivity of 91% and specificity of 97% in differentiating between ONHD and optic disc edema [92]. The scleral canal size can be helpful as it is enlarged in eyes with mild papilledema and narrows as the papilledema resolves; meanwhile, in ONHD it is smaller [55,93]. Using scleral canal size as a tool of differentiation is controversial and challenged by a huge variation in scleral canal size in the general population. PHOMS is a nonspecific marker of axonal congestion and crowding thus cannot be used to discriminate papilledema and ONHD [94,95]. Overall, EDI-OCT provides high clinical significance by enabling clinicians to noninvasively distinguish ONHD from true papilledema, a differentiation critical to avoid unnecessary workups for intracranial hypertension and to appropriately triage patients for urgent neuroimaging or observation.

Kim et al. used enface OCT and OCT-A to compare between buried ONHD and Optic disc edema [95]. They found that on enface OCT, all buried drusen were visualized as well-demarcated hyperreflective kidney-shaped masses, whereas optic disc edema appears as ill-defined boundaries confluent with the retinal nerve fibers. Using OCT-A, 25.0% of the eyes with buried drusen showed a nasal crescentic decrease in radial peripapillary capillary layer vessel density, while 40.3% of the eyes with optic disc edema had nonspecific irregular and focal vessel density decrease around the optic nerve head. Size of ONHD was directly related to the vessel density decrease on OCT-A [95].

## 10. OCT in Morning Glory Anomaly

**Figure 6:**
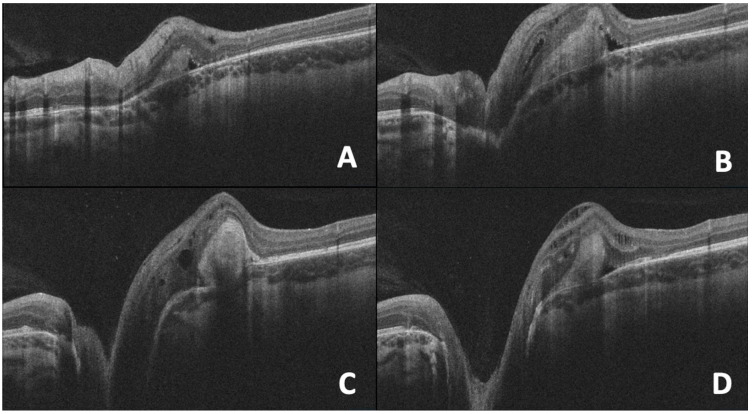
Optical coherence tomography (OCT) cross-sections of the left optic nerve and peripapillary region in a case of morning glory disc anomaly. The images (**A**–**D**) demonstrate an enlarged, excavated optic disc with peripapillary gliosis and associated subretinal and intraretinal fluid consistent with the structural features of this congenital anomaly. Image courtesy of [103].

Morning glory anomaly is characterized by a large funnel-shaped excavation of the optic nerve head and peripapillary retina (Figure 6). The optic disc has a central glial tuft, and the retinal vessels are increased in number and emerging from the disc edge in a radial pattern. The peripapillary area is commonly elevated with a ring of pigmentations, which is possibly a sign of previous detachment. Retinal detachment develops in around a third of the cases of morning glory anomaly. The source of subretinal fluid causing the detachment is controversial, though theories state it could originate from subarachnoid space or vitreous cavity with tractional forces playing a role [96,97]. Morning glory anomaly may develop due to non-closure of the optic fissure, dysgenesis of the terminal optic stalk, or as a primary mesenchymal disorder [98]. Patients with morning glory anomaly should undergo neuroimaging due to associated central nervous system anomalies [99].

OCT shows a large disc with increased cup-to-disc ratio and thinner neuroretinal rim. Retinal nerve fiber layer (RNFL) thickness is greater than normal individuals with disruption of the ISNT rule as the temporal RNFL is the thickest quadrant. In addition, macular thickness is reduced in those cases [100].

Using SD-OCT, Cennamo et al. studied nine eyes with morning glory anomaly [101]. SD-OCT scans revealed glial tissues overlying the optic disc in six eyes and retinal detachment in the conus area of five eyes (four eyes without contractile movements of the optic disc and one eye with contractile movements). In all eyes with retinal detachment, there was an abnormal communication between subarachnoid space and subretinal space. Of the five eyes with retinal detachment, two eyes had retinal breaks but there was no evidence of retinal breaks in three eyes [101]. SS-OCT scans of morning glory anomaly showed the presence of retrobulbar subarachnoid space that directly communicates with the vitreous cavity [102]. Lee et al. evaluated seven eyes with morning glory anomaly and found that all eyes have scleral excavation with epiretinal membrane at its center. Their results helped to subdivide patients with morning glory anomaly into 2 groups: with and without retinal excavation. The younger group without retinal excavation showed intact posterior vitreous attachment to preretinal tissue and peripapillary retinal undulations due to folded retina. Patients with retinal excavation were older in age, and all of them had posterior vitreous detachment [73].

## 11. Myelinated Nerve Fibers

Axons of ganglion cells that form the RNFL are non-myelinated as myelinated nerve fibers that extend from the lateral geniculate body stop at lamina cribrosa. Myelinated nerve fibers are present in less than 1% of the population and are more common in females [104]. Myelinated nerve fibers may be associated with high myopia, amblyopia, and strabismus. A syndrome of myelinated nerve fibers, vitreoretinal degeneration, high myopia, and skeletal malformations has been reported [105].

Clinically, myelinated nerve fibers appear as whitish or greyish lesions with feathery borders following the distribution of RNFL, commonly in the superior part of the optic nerve and are asymptomatic in most cases. On OCT, the RNFL is thickened and hyperreflective in the areas of myelination with shadowing of underlying tissues and preservation of vascular structures [106]. Some studies suggested that myelinated nerve fibers are not connected to RNFL, supporting the theory that the myelination could be a result of abnormal migration of oligodendrocyte-like glial cells to the retina in utero before the lamina cribrosa develops its barrier function or during a period of temporary loss of its function [105,106]. Myelinated nerve fibers appear white on red-free and infrared imaging due to high lipid content of myelin, and dark on autofluorescence and fluorescein angiography due to blocking of normal fundus autofluorescence and fluorescein dye [105,106].

## 12. Melanocytoma

**Figure 7:**
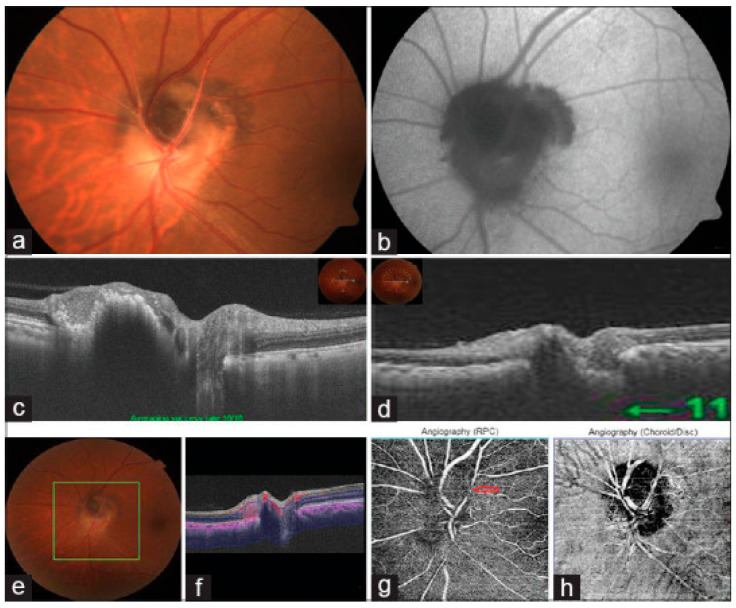
Multimodal imaging of optic nerve head melanocytoma. (**a**) Fundus photograph showing a deeply pigmented lesion involving the optic disc with extension into adjacent retinal and choroidal layers. (**b**) Fundus autofluorescence reveals corresponding hypo-autofluorescence. (**c**,**d**) Spectral-domain OCT demonstrates a dome-shaped hyperreflective mass with posterior shadowing and overlying retinal disorganization; the outer retinal boundary remains visible in portions extending into the choroid. (**e**–**h**) OCT angiography (6 × 6 mm scan) shows increased intrinsic vascularity within the lesion. The radial peripapillary capillary slab (**f**,**g**) demonstrates surface vascular patterns with localized areas of flow attenuation, while the choroidal slab (**h**) shows intrinsic vascular channels and hypo-reflective margins. Image adapted from [112]. This is an open-access article distributed under the terms of the Creative Commons Attribution–NonCommercial–ShareAlike 4.0 License.

Melanocytoma is a benign dark brown to black pigmented lesion that occurs in the optic disc and may involve adjacent retina and choroid. Melanocytoma is often unilateral, non-progressive (in 90% of cases), and occurs at the 6th decade of age. Histologically, melanocytoma is composed of pigmented round to oval nevus cells with benign features. Although most patients with melanocytoma are asymptomatic, visual field defects, mostly in the form of an enlarged blind spot, have been reported in up to 90% of cases. In rare cases, it can cause severe visual loss due to necrosis of the lesion or compressive optic neuropathy [107]. Figure 7 provides multimodal imaging for a case of melanocytoma.

Based on SD-OCT findings, Apinyawasisuk et al. identified two types of optic disc melanocytoma: type one, the typical hyperpigmented lesion with SD-OCT findings of a hyperreflective, disorganized overlying retina and a posterior hyporeflective shadow with an optically clear center, whereas less common is atypical, minimally pigmented lesion with an overlying relatively well-organized retina and lacking a posterior hyporeflective shadow. SD-OCT can also detect complications associated with melanocytoma, such as macular edema, disc edema, or vitreous seeding [108,109].

Due to deeper penetration of SS-OCT, it can better detect peripapillary choroidal invasion of the tumor as thickening, hyperreflectivity, or hyporeflectivity of the choroid [110]. OCT-A shows heterogeneously distributed sparse intrinsic vasculature within the tumor and areas of signal void in the deep retinal layers and choroid, possibly due to the masking effect of the pigments [111].

## 13. Peripapillary and Intrapapillary Pigmentary Structural Optic Nerve Head Changes

### 13.1. A-Grey Crescent

Gray crescent (GC) is considered a normal variant of optic nerve heads that needs to be properly identified during clinical examinations. Shields first described the GC as pigmentations within or on the neuroretinal rim of optic nerve head. The scleral ring defines the border between optic nerve head and peripapillary region, so optic nerve head includes the area inside the scleral ring; meanwhile, the peripapillary region is the area outside the scleral ring, and this is important in the differentiation of GC from peripapillary atrophy. The clinical significance of GC is that the inner edge of the crescent could be mistaken to be the disc border, giving the impression that the pigmented portion of the neuroretinal rim is not within the clinical disc margin, producing the false appearance that the neuroretinal rim is thinner than it actually is [113].

In a series of 29 patients with GC, Shields reported that the mean age of patients was 35 years. GC was more common in individuals with increased skin pigmentation and darker complexion, as 25 of 29 patients were African American, while 4 were white. Moreover, 52% of eyes were myopic and GC was bilateral in 22 of the 29 patients. The crescent was commonly present in the temporal or inferotemporal regions [113]. According to the Reykjavik Eye Study that studied 1012 eyes of a white population, the GC was present in 22% of eyes studied and 58% of those cases were bilateral. It was more common in females, hyperopic patients, and in eyes without PPA. The temporal quadrant was the most common location for GC (37% of eyes) and the supzrior quadrant was the least (1.4%). There was no difference in the prevalence of GC in glaucomatous or non-glaucomatous eyes [114].

When it comes to ophthalmoscopically, two types of GC have been described. Type A gray crescent appears as a slate gray more sloped temporal crescent; meanwhile, type B gray crescent is darker, sharply delineated, and more superficial. Based on a previous study in which high-resolution SD-OCT was used, the investigators characterized the in vivo structure of gray crescent. In Type A crescent, the RPE and Bruch’s membrane were absent and the underlying choroid appeared hyperreflective (analogous to the OCT morphology of the gamma zone of PPA). The underlying dark choroid was directly visible through areas of RPE-Bruch’s membrane loss, giving the type A crescent its dark appearance on fundus examination. On the other hand, the OCT findings of type B gray crescent included RPE-Bruch’s membrane complex clumping or thickening, appearing to be folded upon itself. Thickened RPE with accumulated pigments inside gives the crescent a darker color [115].

On the other hand, Jonas hypothesized that the GC might be due to RPE and Bruch’s membrane extending beyond optic disc borders [116]. Davies et al. [117] and Torres et al. [118] postulated that GC is possibly due to pigmentation of externally oblique border tissue of Elschnig. The border tissue of Elschnig is a collagenous tissue arising from the sclera to join Bruch’s membrane that encloses the choroid and separates it from the retina. When the border tissue of Elshnig forms an obtuse angle with RPE-Bruch’s membrane complex, it is named externally oblique border tissue of Elshnig [119].

Although the exact source of pigmentations causing GC is not determined yet, it could possibly be due to the accumulation of melanocytes migrating from the choroid, and increased incidence of GC among African Americans may support this theory. In addition, the pigmentations could be due to intrapapillary extension of RPE-Bruch’s membrane into the retinal tissues. This can be advocated by the findings of Chauhan et al. who detected that Bruch’s membrane can extend into the optic nerve tissues beyond the border of the disc that is identified clinically [120,121].

### 13.2. B-Peripapillary Atrophy (PPA)

Crescentic PPA is a common clinical finding in normal population and in patients with conditions like glaucoma, high myopia, papillitis, and chorioretinitis [122,123].

Peripapillary atrophy is typically a lighter colored, well-demarcated crescent-shaped area on the temporal side of the optic nerve. PPA develops due to choroidal thinning and disruption of RPE around the optic disc. PPA can be clinically divided into a central beta (β) zone and peripheral alpha (α) zone. Alpha-zone is characterized by irregular hypopigmentation and hyperpigmentation due to thinning and interruption of the RPE and underlying choroid. On the contrary, β-zone is associated with marked atrophy and loss of the RPE with intact Bruch’s membrane and underlying choriocapillaris, allowing good visibility of the large choroidal vessels and sclera [124].

Jonas and colleagues described two additional zones: gamma zone and delta zone. Gamma zone was described as an area of parapapillary sclera without overlying choroid, Bruch’s membrane, and deep retinal layers. Delta zone is an area with a length of ≥300 mm in the central part of gamma zone in which blood vessels of at least 50 mm diameter were not present. Both zones are related to axial globe elongation and independent of glaucoma [125]. Recently, focal gamma zone has been reported to be possibly associated with glaucoma development while the conventional gamma zone is not [126].

Manjunath et al. evaluated 103 eyes with PPA using high-definition SD-OCT and described six OCT characteristics of PPA: RPE disruption (82% of eyes), RNFL thickening in the form of plaques (45%), RPE and partial photoreceptor loss (29%), abnormal retinal sloping (23%), retinal thinning (19%), and intraretinal cystic changes (2%). Total retinal thickness was thicker by 15% in normal eyes compared with eyes with PPA [127].

The relationship between PPA and glaucoma development and progression has been of interest as it may help clinicians to closely follow up patients at higher risk of visual function deterioration. Beta zone is commonly associated with glaucoma and the size of α and β zones are larger in glaucomatous eyes than in normal eyes [124]. Some studies suggested that the area of PPA increases with the progression of glaucomatous optic nerve damage and worsening of the visual field. The location of the largest β-zone PPA typically correlates with the region of the most rapid VF progression [128,129]. Teng et al. evaluated 245 eyes with glaucoma retrospectively in correlation with their visual fields and reported that eyes with β-PPA progressed more rapidly (−0.84 ± 0.8 dB/year) than eyes lacking β-PPA (−0.51 ± 0.6 dB/year; *p* < 0.01). A significant positive correlation between thinner central corneal thickness (CCT) and presence of β-PPA was also present [130].

On the contrary, in another study that followed up patients with ocular hypertension (OHT) progressing to glaucoma and OHT patients that did not progress, their results suggested that there was no difference in baseline measurements of the area of β-PPA between the two groups and the increasing size of β-PPA did not change significantly over the follow-up period between the two groups [131].

In contrast to glaucomatous optic neuropathy, non-glaucomatous optic nerve damage, as in non-arteritis anterior ischemic optic neuropathy, does not lead to an enlargement of PPA [132].

In highly myopic eyes, PPA is more common than in emmetropic eyes and is present in approximately 20% of high myopes [133]. The ultrastructure of myopic crescents is similar to β-zone of PPA, which was confirmed by the SD-OCT findings of these crescents showing complete loss of the RPE layer and a partial loss of the photoreceptor layer. As myopia develops, the axial length increases in size, causing retinal stretching, thinning, and pulling of the RPE and choroid away from the optic disc [134].

### 13.3. C-Peripapillary Halo

Peripapillary halo (PPH) is a pigmentary retinal change encircling the optic nerve head in normal eyes and eyes with different pathologies. PPH is seen outside the border of optic nerve disc, which differentiates it from the intrapapillary optic nerve gray crescent. “Halo Glaucomatous” is a PPH surrounding the ONH that develops in advanced cases of glaucoma [135].

PPH can exist in high myopic eyes, but it is less common than crescentic PPA. Nokana et al. analyzed the characteristics of highly myopic crescentic PPA and PPH (PPH was defined as atrophy with circumferential extent > 9 clock hours around optic nerve head in that study) using cSLO (confocal scanning laser ophthalmoscope) fundus images and SD-OCT. In patients with PPH compared to crescentic PPA, mean age was more by 10 years, area of atrophy was wider, distance from foveal center to temporal margin of atrophy was shorter, and retinal layers terminated further from the temporal edge of atrophy [136].

PPH can occur in patients with posterior uveitis, as in birdshot chorioretinopathy (BSCR), where lesions affect the peripapillary retina and choroid. In B-scans of SS-OCT across the area of PPH, it was reported that the area of PPH correlated with RPE-Bruch’s membrane thinning and hyperreflectivity of the underlying choroid. So, PPH in BSCR could be considered as a circumferential form of alpha zone of PPA [137].

Based on these OCT findings, it was hypothesized that PPH may develop due to swelling of the optic nerve head secondary to papillitis, causing stretching and thinning of the retinal layers attached to the border tissue of Elschnig. After the resolution of optic nerve head edema, the retinal layers are no longer fully attached to the disc border and have an atrophic circumferential halo. PPH is of crucial clinical significance as the presence of PPH in BSCR patients may provide a clinical indicator of previous episodes of papillitis [137].

## 14. Limitations

This study has several important limitations related both to the methodology and the use of OCT as an imaging modality. First, the retrospective nature of the study introduces inherent selection bias. Only patients with available and interpretable OCT imaging were included in this study, potentially excluding those with poor-quality scans or atypical presentations. Additionally, there was no standardized OCT acquisition protocol across patients. Thus, variations in scan resolution, centering, and signal strength may have impacted the accuracy and consistency of RNFL and GC-IPL measurements.

Device heterogeneity and protocol variability are also significant limitations in the interpretation and comparability of OCT studies across diseases. Differences in hardware, software algorithms, and analysis protocols among OCT devices result in systematic discrepancies in quantitative measurements such as retinal thickness and vessel density, which can exceed clinically acceptable limits and raise concerns when directly comparing or pooling data from different instruments [138,139]. For example, studies have shown poor agreement in retinal nerve fiber layer thickness and macular volume measurements between devices, even when using standard protocols, due to differences in segmentation algorithms and assumed ocular biometry [138,139,140]. Another limitation in comparing different studies that utilize OCT is protocol variability. This includes scan patterns, image scaling, and analysis methods, all of which can lead to inconsistent reporting and interpretation of disease biomarkers. These limitations highlight the need for standardized acquisition and analysis protocols to enable reliable cross-study comparisons, facilitate multicenter trials, and support the development of normative databases that can be utilized irrespective of device/protocol heterogeneity.

Furthermore, image review and interpretation were not performed in a masked fashion, raising the risk of observer bias, especially in assessing qualitative features such as peripapillary folds, shape deformations, or optic disc drusen. While the study emphasized the diagnostic value of structural OCT parameters, it did not integrate functional measures such as visual field data to correlate structure–function relationships. Moreover, the lack of longitudinal follow-up in most cases prevented analysis of temporal changes in OCT parameters, limiting the ability to evaluate progression or treatment response.

Additionally, artifacts and limited tissue penetration are critical limitations of OCT with direct clinical implications. Artifacts such as segmentation errors, decentration, and operator-dependent acquisition issues are prevalent, particularly in eyes with retinal pathology, high myopia, or media opacities, and may not always be apparent on standard printouts [141,142,143,144]. These artifacts can result in misdiagnosis or underestimation of lesion extent, leading to false-positive or false-negative assessments of disease progression, especially in glaucoma and macular diseases [142,143]. Penetration limits, particularly in the presence of dense media opacities or highly reflective lesions, may obscure deeper structures, further increasing the risk of missing clinically significant pathology [59,141]. Clinicians must systematically review raw B-scan images and remain cautious of artifacts to avoid errors in therapeutic decisions.

It is also important to recognize that existing OCT normative databases may not fully capture variations related to age, axial length, or ethnicity, which can influence retinal layer measurements and occasionally lead to misclassification in atypical populations. Several studies have emphasized the need for population-specific reference data to improve diagnostic accuracy across diverse groups [145,146,147].

In real-world settings, imaging children and non-cooperative patients also presents distinct challenges, as motion artifacts, limited fixation, and poor compliance can compromise scan quality and repeatability. The need for sedation or handheld OCT devices often arises in pediatric or neurologically impaired populations, which may restrict the availability of longitudinal data. Continued advances in faster acquisition speeds, eye-tracking, and portable OCT systems are helping to overcome these limitations and improve clinical applicability.

Finally, the normative data used for comparison may not have accounted for patient-specific factors such as age, axial length, or ethnicity, all of which can influence OCT measurements and complicate interpretation in borderline or atypical cases.

## 15. Future Directions

OCT imaging continues to rapidly evolve as technology advances. With the rise of artificial intelligence (AI), OCT is increasingly being integrated into the development of automated diagnostic tools. A recent study demonstrated the diagnostic potential of unsegmented 3D OCT data in differentiating optic disc edema etiologies, including idiopathic intracranial hypertension (IIH) and non-arteritic anterior ischemic optic neuropathy (NAION), achieving high external validation accuracy (90.1%) [148]. Similarly, in glaucoma, machine learning models using features extracted from segmented OCT B-scans have achieved near-perfect diagnostic performance (AUC: 0.99), incorporating multidimensional parameters like shape, reflectivity, and texture to move beyond traditional thickness-based metrics [149]. AI has also been used for quantitative OCT biomarkers, which are powerful tools for transforming how retinal and optic nerve diseases are predicted and managed. By providing objective and reproducible measurements of retinal structure, these biomarkers allow clinicians to move beyond qualitative assessment and more towards data-driven, personalized care. AI models can use these parameters to stratify risk, monitor disease progression, and inform therapeutic decisions. For instance, a recent machine learning study in geographic atrophy showed that automatically quantified OCT features, such as retinal pigment epithelium loss and photoreceptor degeneration, predict both standard visual acuity (r^2^ = 0.40; MAE = 11.7 ETDRS letters) and low-luminance visual function (r^2^ = 0.25; MAE = 12.1 ETDRS letters) with clinically meaningful accuracy, highlighting their potential for individualized management [150]. As AI becomes further integrated into imaging workflows, quantitative OCT biomarkers are expected to support earlier diagnosis, tailored treatment strategies, and standardized outcome measures for clinical trials [151]. Looking forward, the application of AI may not only aid in early diagnosis but also in individualized risk stratification, longitudinal monitoring, and prediction of visual outcomes.

In parallel, innovations in OCT hardware are expanding its reach beyond traditional clinical environments [152]. Handheld OCT systems have transformed pediatric imaging by enabling non-contact, high-resolution retinal assessment in neonates and infants. These devices have revealed retinal features previously undetectable with traditional ophthalmoscopy such as infant macular edema and perifoveal vascular development [153]. In surgical settings, intraoperative OCT has been integrated into surgical microscopes, providing real-time feedback during ophthalmic procedures such as retinal membrane peeling or glaucoma surgery. This intraoperative imaging enhances surgical decision-making by revealing tissue planes, confirming surgical endpoints, and minimizing unnecessary manipulation [153]. Future directions include improving ergonomics and reducing workflow disruption through robotic integration and automated image registration.

Extending beyond clinic and operating room settings, at-home OCT systems represent a promising future for patient care and monitoring. An at-home device can enable daily self-imaging and offer a promising solution for longitudinal monitoring of retinal diseases like neovascular AMD. Studies have shown high patient adherence and strong agreement between home and clinic-acquired scans, supporting the feasibility of remote disease surveillance [154]. Integration with AI tools like the Notal OCT Analyzer further enhances this approach by enabling automated detection of retinal fluid, which may facilitate earlier treatment decisions and reduce the risk of vision loss.

Lastly, the integration of AI with OCT-A represents a significant step forward in neuro-ophthalmic imaging. Bunod et al. evaluated the diagnostic performance of a deep learning system using OCT-A images to differentiate glaucoma, NAION, and healthy controls. The model that combined macular superficial capillary plexus (SCP) and radial peripapillary capillary (RPC) plexus images achieved a mean area under the receiver operating characteristic curve (AUC) of 0.94 (95% CI 0.92–0.96) for glaucoma, 0.90 (95% CI 0.86–0.94) for NAION, and 0.96 (95% CI 0.96–0.97) for healthy controls. These metrics indicate high diagnostic accuracy for all three groups, with the model performing best in distinguishing healthy controls and glaucoma, and slightly lower but still robust accuracy for NAION [155]. A review by Hormel et al. further emphasizes that AI is rapidly advancing OCT-A’s clinical utility by enabling automated segmentation, quantification of vascular parameters, and disease detection. AI integration with OCT-A provides objective, reproducible analysis of microvascular changes, which is especially valuable for early diagnosis, monitoring progression, and distinguishing between overlapping neuro-ophthalmic entities. However, the review notes that most AI applications in OCT-A remain in the research phase, and more studies are needed to develop more robust machines [156].

## 16. Conclusions

OCT imaging has transformed neuro-ophthalmic practice, and with ongoing technological advancements, its applications are set to expand further. We have outlined its role in diagnosing, monitoring, and predicting outcomes for common optic nerve diseases. It is worth mentioning that computer-generated data must be critically evaluated for potential errors or artifacts and interpreted within the clinical context. As OCT technology evolves, it promises to deepen our understanding of optic nerve disease pathophysiology in both research and clinical settings.

## Figures and Tables

**Table 1:** Summary of key optical coherence tomography (OCT) findings and clinical insights in optic nerve head disorders.

Condition	Key OCT Findings	Key Learning Point
1. Glaucoma	RNFL thinning (especially inferior/superior quadrants); GCIPL loss	OCT and OCT-A provide objective assessment of structural and vascular damage. RNFL and GCIPL analyses enable early detection and progression monitoring, while reduced peripapillary capillary density correlates with disease severity.
2. AION (NAION/AAION)	Acute: RNFL thickening. Chronic: RNFL thinning and GCIPL loss	Sequential OCT changes reflect ischemic damage, acute swelling followed by RNFL thinning and GCIPL loss. OCT-A shows greater vessel density and tortuosity loss in AAION than NAION, reflecting more severe microvascular damage.
3. Optic Neuritis (MS/NMO)	RNFL thickening acutely → Early GCIPL loss	OCT complements MRI by detecting chronic neuroaxonal loss and subclinical progression in MS-related optic neuritis, which MRI may miss.
4. Neuroretinitis	Subretinal and intraretinal fluid; macular star formation	OCT detects early neuroretinitis changes before macular star formation, improving diagnostic accuracy.
5. Papilledema	Diffuse peripapillary RNFL thickening in all quadrants with optic nerve head elevation on B-scan.	RNFL thickening, especially in the nasal quadrant, supports true optic disc edema; OCT-A vessel density correlates with disease severity in IIH.
6. Pseudopapilledema	Normal or only mildly increased average RNFL thickness without diffuse quadrant thickening or true disc elevation.	OCT helps differentiate pseudopapilledema from papilledema, but interpretation must consider refractive error, pediatric norms, and device variability.
7. Optic Pit	Deep excavation of the optic disc with lamina cribrosa defect, herniation of nerve tissue, and associated serous macular detachment or intraretinal schisis.	Advanced OCT (EDI and SS-OCT) enables full visualization of pit morphology and fluid dynamics, improving diagnosis and monitoring of OP maculopathy.
8. Optic Nerve Head Drusen	Signal-poor core with hyperreflective margins above the lamina cribrosa; best visualized on EDI-OCT.	EDI-OCT is the gold standard for drusen detection, distinguishing ONHD from papilledema and revealing detailed drusen morphology.
9. Morning Glory Anomaly	Large, funnel-shaped excavation of the optic disc with peripapillary gliosis, subretinal fluid, and abnormal communication with the subarachnoid space.	OCT reveals characteristic disc excavation and associated retinal detachment, aiding differentiation from other congenital optic nerve anomalies.
10. Myelinated Nerve Fibers	Localized RNFL thickening with hyperreflectivity and shadowing of underlying layers.	OCT confirms myelination within the RNFL and helps differentiate it from true optic disc edema.
11. Melanocytoma	Dome-shaped hyperreflective lesion with posterior shadowing and overlying retinal disorganization; SS-OCT may show choroidal invasion.	OCT and OCT-A help differentiate melanocytoma from malignant lesions by revealing its structural depth, intrinsic vascularity, and benign features.

Summary of characteristic OCT and OCT angiography (OCT-A) features across common optic nerve pathologies. OCT = optical coherence tomography; OCT-A = optical coherence tomography angiography; RNFL = retinal nerve fiber layer; GCIPL = ganglion cell-inner plexiform layer; AION = anterior ischemic optic neuropathy; AAION = arteritic anterior ischemic optic neuropathy; NAION = non-arteritic anterior ischemic optic neuropathy; MS = multiple sclerosis; NMO = neuromyelitis optica; IIH = idiopathic intracranial hypertension; EDI-OCT = enhanced depth imaging optical coherence tomography; SS-OCT = swept-source optical coherence tomography; ONHD = optic nerve head drusen.

## Data Availability

Data are available upon requests to the corresponding author.
